# Controls on Interspecies Electron Transport and Size Limitation of Anaerobically Methane-Oxidizing Microbial Consortia

**DOI:** 10.1128/mBio.03620-20

**Published:** 2021-05-11

**Authors:** Xiaojia He, Grayson L. Chadwick, Christopher P. Kempes, Victoria J. Orphan, Christof Meile

**Affiliations:** a Department of Marine Sciences, University of Georgia, Athens, Georgia, USA; b Division of Geological and Planetary Sciences, California Institute of Technology, Pasadena, California, USA; c The Santa Fe Institute, Santa Fe, New Mexico, USA; Oregon State University

**Keywords:** syntrophy, FISH-nanoSIMS, activation loss, anaerobic oxidation of methane, conductive network density, conductivity, direct interspecies electron transfer, electron conduction, ohmic resistance, spatial statistics, stable isotope probing, syntrophy

## Abstract

About 382 Tg yr^−1^ of methane rising through the seafloor is oxidized anaerobically (W. S. Reeburgh, Chem Rev 107:486–513, 2007, https://doi.org/10.1021/cr050362v), preventing it from reaching the atmosphere, where it acts as a strong greenhouse gas. Microbial consortia composed of anaerobic methanotrophic archaea and sulfate-reducing bacteria couple the oxidation of methane to the reduction of sulfate under anaerobic conditions via a syntrophic process. Recent experimental studies and modeling efforts indicate that direct interspecies electron transfer (DIET) is involved in this syntrophy. Here, we explore a fluorescent *in situ* hybridization-nanoscale secondary ion mass spectrometry data set of large, segregated anaerobic oxidation of methane (AOM) consortia that reveal a decline in metabolic activity away from the archaeal-bacterial interface and use a process-based model to identify the physiological controls on rates of AOM. Simulations reproducing the observational data reveal that ohmic resistance and activation loss are the two main factors causing the declining metabolic activity, where activation loss dominated at a distance of <8 μm. These voltage losses limit the maximum spatial distance between syntrophic partners with model simulations, indicating that sulfate-reducing bacterial cells can remain metabolically active up to ∼30 μm away from the archaeal-bacterial interface. Model simulations further predict that a hybrid metabolism that combines DIET with a small contribution of diffusive exchange of electron donors can offer energetic advantages for syntrophic consortia.

## INTRODUCTION

Anaerobic oxidation of methane (AOM) coupled to sulfate reduction (SR) is a globally important process commonly catalyzed by a consortium of anaerobic methanotrophic archaea (ANME) and sulfate-reducing bacteria (SRB) (1–4). AOM in marine sediments reduce emissions of the potent greenhouse gas methane (5) to the overlying water and the atmosphere. Due to the role of methane in atmospheric radiative forcing (6), it is important to understand the processes and mechanisms involved in AOM. Recent studies provide evidence that supports direct extracellular electron transfer, for example, in single (7, 8)- and mixed (9)-species *Geobacter* biofilms. Furthermore, direct interspecies extracellular electron transfer (DIET) has been observed in cocultures (10, 11) and microbial aggregates (12–16). There is also a growing body of evidence that DIET also takes place between methanotrophic archaea and syntrophic sulfate-reducing bacteria in AOM consortia (17–19), where it serves as an effective transport mechanism over long spatial distances (17). It overcomes limitations inherent in the diffusive exchange of dissolved electron-carrying molecules (mediated interspecies electron transfer, or MIET) that lead to the build-up of reaction products and the subsequent shutdown of metabolic activity (19, 20).

DIET is thought to occur through a variety of mechanisms, including direct contact between cells (21), through electrically conductive pili (10, 11, 13–15) and/or extracellular cytochromes (11, 15, 16). Genomic and transcriptomic data of enrichments with different types of AOM consortia (ANME-1a/HotSeep-1, ANME-1a/Seep-SRB2, and ANME-2c/Seep-SRB2) revealed that genes encoding flagella or type IV pili, and/or surface-bound or extracellular *c*-type cytochromes, were highly expressed (22). Notably, ANME-2 genomes encode large multiheme cytochromes containing putative S-layer domains (17) thought to be analogous to the Gram-negative porin-cytochrome conduits in that they can be used for electron egress through the outermost cell layer (23). Observations using transmission electron microscopy (TEM) showed staining consistent with heme-rich areas and pilus/wire-like structures in the intracellular space in AOM consortia (17, 18, 22). These features suggest that DIET is the principal mechanism of sulfate-dependent AOM. While this hypothesis awaits direct experimental confirmation or indirect support through measurements that show the potential for conduction within the aggregates and is hampered by a lack of any pure cultures of microorganisms carrying out this metabolism, modeling efforts indicated that DIET can support cell-specific AOM rates and archaeal activity distributions that were consistent with observations from single-cell resolved fluorescent *in situ* hybridization-nanoscale secondary ion mass spectrometry (FISH-nanoSIMS) analyses (19).

Recently, a finite distance over which extracellular electron transport sustains metabolic activity was documented in biofilms of Geobacter sulfurreducens (24). These results suggest that the extent to which conductive biomolecules can support optimal cell growth away from an electrode surface is limited (24, 25). Using a similar experimental approach, a drop in activity with distance between electron donors (archaea) and acceptors (bacteria) was not observed in AOM aggregates (17–19). However, the size of the microbial aggregates analyzed was much smaller than the *Geobacter* biofilm thickness, leading to short separation distances between the syntrophic partners within the aggregates (17). In this study, we target exceptionally large aggregates (radius, ∼20 μm) in which bacteria and archaea were spatially segregated. We measured and analyzed the metabolic activity of individual cells using FISH-nanoSIMS. Measurements of ^15^NH_4_^+^ incorporation are then used to validate a reactive transport model. Simulation results consistent with our empirical observations form the basis for three key novel aspects of this work. First, we investigate the mechanisms of potential losses associated with direct extracellular electron transport by accounting for ohmic resistance and activation loss that ultimately limits metabolic activity away from an archaeal-bacterial interface, an effect not apparent in small or well-mixed aggregates we have reported on earlier (17, 19). Second, we investigate the potential for environmentally sourced electron donors used by the SRB, partially decoupling archaeal methanotrophy and bacterial sulfate reduction. Third, we consider the advantages of a hybrid DIET-MIET mechanism that can offer energetic benefits allowing for balanced microbial energetics for both syntrophic partners, particularly for large aggregates.

## RESULTS AND DISCUSSION

### Large, segregated aggregates display significant spatial variation in cellular activity.

Previous experimental work measuring the activity of individual cells in syntrophic ANME-SRB aggregates demonstrated a lack of significant correlation between cellular activity and distance to syntrophic partner over short distances (a few cell diameters [17]). These observations were sufficient to rule out molecular diffusion as the major mechanism of electron transfer between the two partners but were limited in their spatial extent due to relatively small aggregate size as well as the complex three-dimensional structure of many AOM consortia that made it difficult to confidently assign distances to nearest partners that may lie above and below the plane when analyzing single two-dimensional cross-sections. We have occasionally observed exceptionally large AOM consortia in nanoSIMS analyses where significant variations in activity appear to be related to distance from their nearest partner (for example, see Fig. S1 at https://doi.org/10.6084/m9.figshare.13536086.v2). While these previous observations suggested that cellular activity is correlated with distance to nearest syntrophic partner over large distances, it was not possible to determine a precise magnitude of the activity gradients without additional information about the three-dimensional aggregate structure.

**FIG 1:**
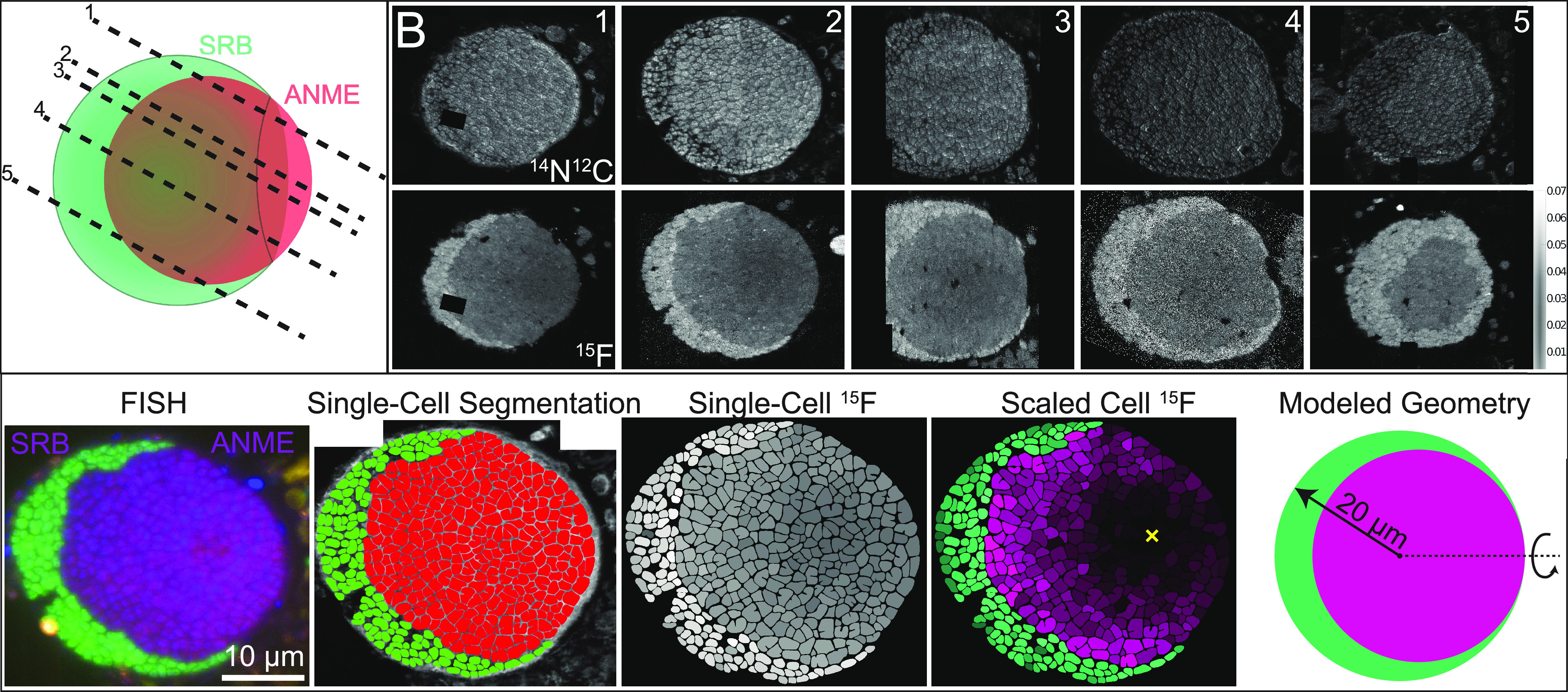
Overview of AOM consortium structure, nanoSIMS data acquisition, analysis, and model geometry. (A) Cartoon of AOM consortium structure based on FISH-nanoSIMS observations of five parallel sections corresponding to dashed lines. (B) Five parallel sections highlighted in panel A analyzed by nanoSIMS. Top row, raw ^14^N^12^C^−^ secondary ion counts illustrating the position of cells. Bottom row, fractional abundance of ^15^N calculated as ^15^N^12^C^−^/(^15^N^12^C^−^+^14^N^12^C^−^), all scaled to the same intensity. Note sulfate-reducing bacteria (SRB) assimilate significantly more ^15^N, on average, than their ANME-2 counterparts, as has been previously shown (17). (C) Illustration of nanoSIMS data extraction and modeled geometry. From left to right, FISH image indicating phylogenetic identity of cells (green, general bacterial probe [Eub338mix]; red, ANME-2b-specific probe [ANME-2b-729]; blue, DNA stain [DAPI]); segmentation image showing SRB and ANME cells manually segmented based on observation of FISH and nanoSIMS data; individual segmented cells shaded by their total ^15^N fractional abundance; SRB and ANME cells scaled by minimum and maximum values within the population; and illustration of modeled aggregate geometry (the dashed line represents axis of rotation). The yellow X marks the approximate minimum ANME cell activity. Note that additional sections were visually inspected to help verify aggregate structure. Only those analyzed by nanoSIMS analysis are shown. This figure contains tiled images that were stitched together to make these composite images. Black regions within the image are places where the square tiles did not overlap.

To overcome these challenges, we cut and analyzed parallel sections through a large, well-segregated ANME-2/SRB consortium after ^15^NH_4_^+^ stable isotope probing, allowing us to roughly reconstruct the spatial distribution of both partners across the entire consortia (Fig. 1A and B). Two features of this >50-μm AOM consortium made it ideal to study. First, the spatial organization of the syntrophic partnership is simple and well defined, with no incursions of bacteria into the ANME-dominated interior of the aggregate. Second, the bacteria form a crescent around the archaeal core instead of a complete shell. Had the bacteria formed a complete shell, there would be perfect correlation between ANME distance to nearest syntrophic partner and distance to the surface of the aggregate, making these two potential controls on cellular activity difficult to disentangle. With a crescent geometry, however, some ANME can be found at the surface of the consortia closest to the surrounding environment and at great distance from the nearest SRB, allowing us to distinguish between the effect of syntrophic distance and distance to the environment that supplies the growth substrates CH_4_ and SO_4_^2−^ and the tracer ^15^NH_4_^+^. Since the minimum ANME activity was observed to be near the aggregate surface, far from the SRB, we conclude that distance to partner is more significant than substrate limitation (Fig. 1C). This finding is consistent with the measurements in the second large aggregate we observed, one with a slightly less segregated distribution of archaea and bacteria, as shown in Fig. S1 at https://doi.org/10.6084/m9.figshare.13536086.v2.

### A unifying model across aggregate size.

**FIG 2:**
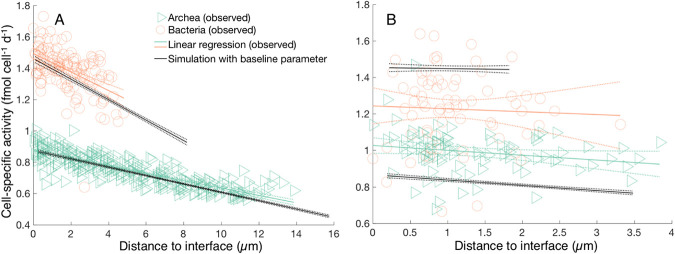
Measured and modeled cell-specific activity in aggregates with a radius of 20 μm (A; this study) and 5 μm (B; 17), plotted against their distance from the closest syntrophic partner (interface). Data were fitted using linear regression with 95% confidence intervals. Note that the cell-specific rate constants were not retuned to match the activities in the small aggregate.

Observations of ^15^N incorporation in single cells from a section cut approximately normal to the ANME-SRB interface revealed a decrease in the anabolic activity of both ANME and SRB with increasing distance to their nearest syntrophic partners (Fig. 2A). This effect was highly significant and explained large portions of the variability of cellular activity in the two populations, with a slope of −0.0238 ± 0.0009 fmol cell^−1^ day^−1 ^μm^−1^ (*R*^2^ = 0.69) and −0.0594 ± 0.0083 fmol cell^−1^ day^−1 ^μm^−1^(*R*^2^ = 0.27) for archaea and bacteria, respectively (Fig. 2A). Our base model, in which 92.5% of the electrons produced in the oxidation of CH_4_ are transferred to the bacteria via DIET and 7.5% of the electrons are transferred via MIET, provides the best fit of the activities observed in aggregates across a wide range of aggregate sizes (Fig. 2). Cell-specific activities decrease slightly with increasing distance from the nearest syntrophic partner in a simulated 20-μm radius aggregate, with slopes of −0.0267 ± 0.0004 fmol cell^−1^ day^−1 ^μm^−1^ (*R*^2^ = 0.9954) and −0.0653 ± 0.0017 fmol cell^−1^ day^−1 ^μm^−1^ (*R*^2^ = 0.9936) for archaeal and bacterial activity, respectively (Fig. 2A). One-way analysis of covariance (ANCOVA) revealed that the slopes and intercepts of the regressions of model results and of observational data do not differ significantly, with *P *values of 0.30 and 0.71 for archaea and bacteria, respectively. Simulations for a small aggregate with the identical model parameterization retained good agreement between observed and modeled metabolic activity patterns (Fig. 2B), with a *P *value of 0.96 for both archaea and bacteria compared to observations.

### How far apart can ANME and SRB cells be and remain active in AOM consortia?

**FIG 3:**
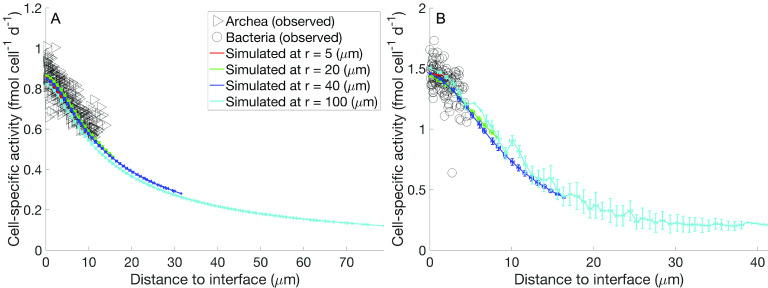
Cell-specific activity versus distance from syntrophic partner for archaea (A) and bacteria (B). For a wide range of aggregate sizes (*r*_agg_ = 5, 20, 40, and 100 μm), the simulated activity distribution is similar and depends on the distance from the interface between archaea and bacteria.

The metabolic activity of syntrophic AOM aggregates can be limited by the availability of electron donors and acceptors, as reflected by the thermodynamics (equation 6) of the overall reactions (termed Rxn3 and Rxn4 and described in Materials and Methods). Here, we investigate the internal and external constraints that potentially limit the metabolic activity within the context of the observed aggregate arrangement. All archaeal and bacterial cells remained active over a wide range of aggregate sizes in our model simulations (Fig. 3); however, the simulated activity of individual cells did decrease with increasing distance from their syntrophic partners. This effect is observed in model simulations for both archaea (Fig. 3A) and bacteria (Fig. 3B) and is slightly steeper for the latter. The shape and magnitude of the activity decrease curve were nearly identical between aggregates of different sizes, highly consistent with what we observed with anode-respiring G. sulfurreducens biofilms of different thicknesses under high and low anode potentials (24). We included in our model simulations segregated aggregates with radii of up to 100 μm (same spatial arrangement as that shown in Fig. 1C). In strongly segregated AOM aggregate and over sufficiently long distances, cell activity decreases with distance to the syntrophic partner even with electron transfer via DIET. Cellular activities in strongly segregated large aggregates experienced a >70% drop in activity as separation distances increase to 15 μm for bacteria and to 30 μm for archaea (Fig. 3). Thus, DIET allows for much bigger clusters than can be supported with MIET alone.

Simulations including molecular diffusion (MIET) of potential syntrophic intermediates, such as intermediate in addition to DIET, revealed that metabolic activity could become severely limited with large separation distances between partners (aggregate size [*r*_agg_], 60 μm; see Fig. S9 at https://doi.org/10.6084/m9.figshare.13536086.v2), even though MIET only accounted for 7.5% of the electron transfer from archaea to bacteria. It is noted that even at this size extreme, the mass transport of substrates and metabolites, including CH_4_, SO_4_^2−^, HS^−^, H^+^, and HCO_3_^−^, was not limiting due to the relatively high concentrations of methane and sulfate at the outer environmental boundary, varying by a factor of less than 1%, except for HS^−^, which varies by 10% across the aggregate (data not shown). These results suggest that this distance-dependent cellular activity pattern is a critical factor determining the size of monospecies clusters within AOM consortia. Thus, larger aggregates would be expected to have a more interspersed distribution of archaeal and bacterial partners to maintain high levels of single-cell activity or, once a segregated aggregate size limit is reached, larger consortia then separate into two or transform into a larger clustered morphology as bacteria grow into the archaeal core (26).

### What controls the spatial distribution of activity?

**FIG 4:**
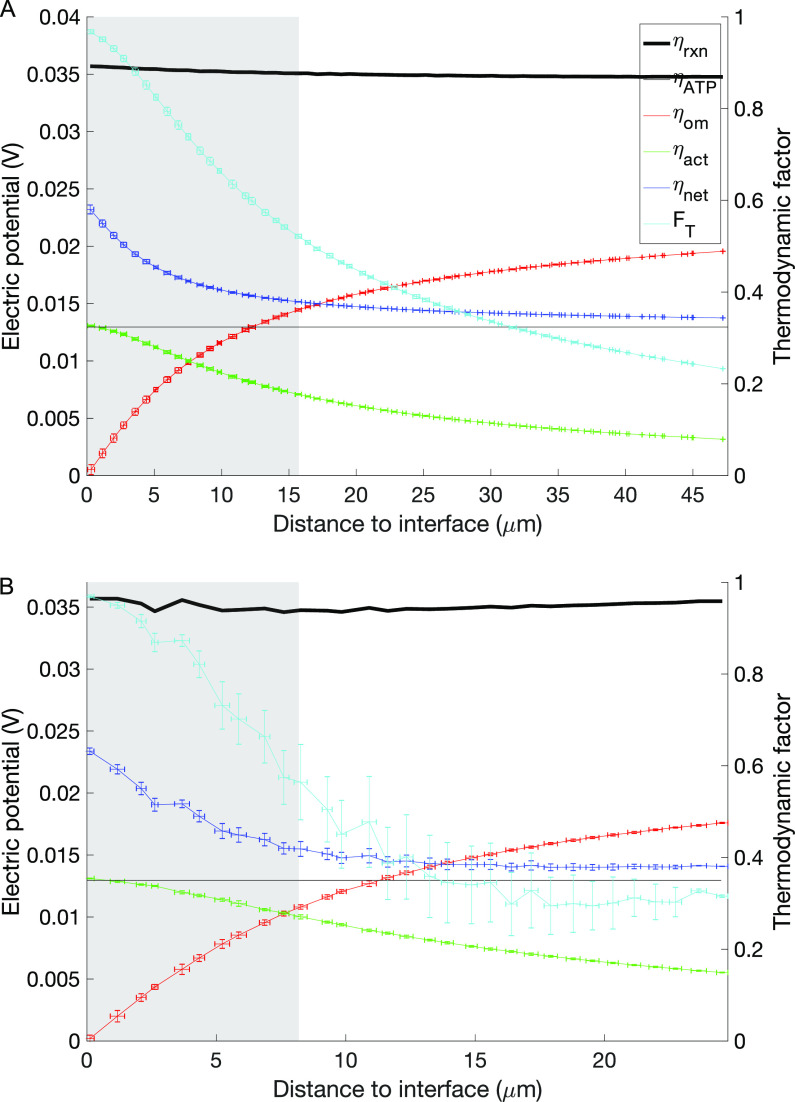
Factors controlling cell activity as a function of the distance from the archaeal-bacterial interface at aggregate radius of 60 μm for archaea (A) and bacteria (B). The left axis reflects electric potential for activation loss (η_act_), ohmic resistance loss (η_om_), net available potential (η_net_), potential from reaction (η_rxn_), and minimum potential required for ATP synthesis (η_ATP_). The right axis reflects the thermodynamic factor, *F_T_*. The shaded areas highlight the range of distances encountered in the observed aggregate with a 20-μm radius (Fig. 1).

The spatial variation of the cell metabolic activity was found to depend on the usable electric potential (η_net_), which is set by the available energy from the reaction (at approximately 0.0357 V for archaea and bacteria) minus the effect of losses. The activation loss was the main contribution to potential losses at distances of approximately ≤8 μm to the partner interface, while ohmic losses were important at larger distances (Fig. 4). This pattern was observed for both archaeal and bacterial cells. Activation loss was maximal at the archaeal-bacterial interface, with a value of 0.013 V, and decreased away from the archaeal-bacterial interface. In contrast, ohmic resistance loss increased from 0 to ∼0.02 V as the distance from the archaeal-bacterial interface increased, leading to a maximum total potential loss at a value of ∼0.023 V for archaea and bacteria. As the net available potential (η_net_) approaches the minimum potential required for ATP synthesis (∼0.013 V; equation 6), metabolic rates decrease due to energetic limitations, as indicated by the thermodynamic factor, *F_T_*, approaching 0 (Fig. 4).

**FIG 5:**
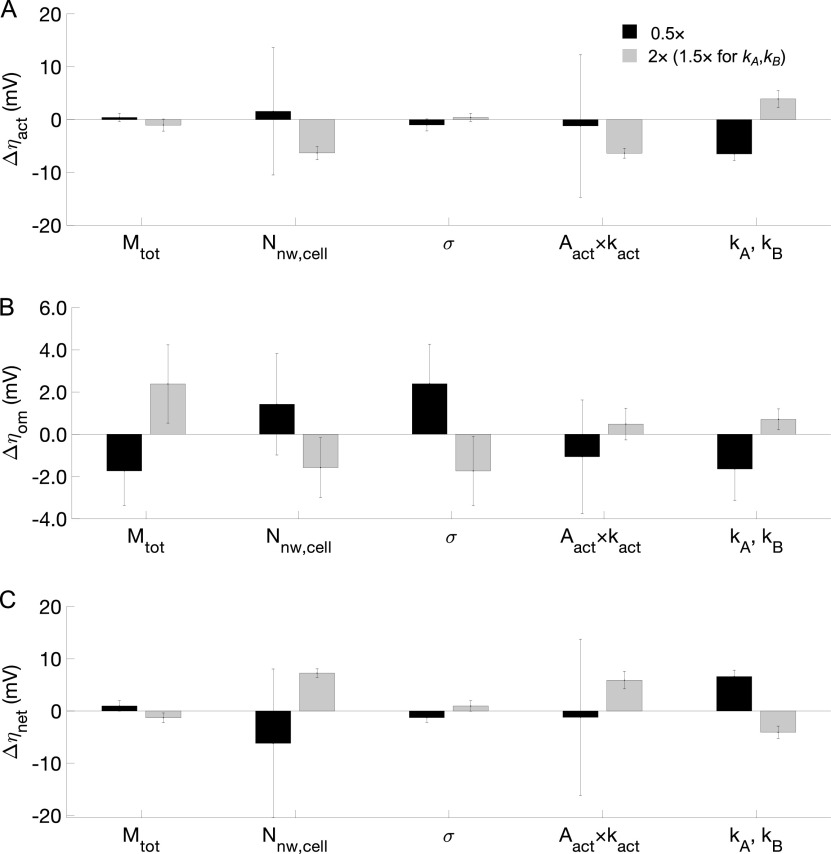
Changes of activation loss, Δ*η*_act_ (A), ohmic resistance loss, *Δη*_om_ (B), and net available potential, Δη_net_ (C), due to a change in total redox active molecules (*M*_tot_), number of conductive connections (*N*_nw,cell_), conductivity (σ), cell redox active factor (*k*_act_×*A*_act_), and cell rate constants (*k_A_* and *k_B_*). Error bars reflect that the impact is not exactly constant with distance for archaeal-bacterial interface (see Fig. S11 to S13 at https://doi.org/10.6084/m9.figshare.13536086.v2).

Voltage losses depend on a number of factors, including the concentration of redox-active molecules (*M*_tot_), conductive network density (*N*_nw,cell_), its conductivity (*σ*), cell surface redox activation factor (*k*_act_*×A*_act_), and cell rate constants (*k_A_*, *k_B_*). Activation loss was strongly impacted by *k_A_*, *k_B_*, *N*_nw,cell_, and *k*_act_*×A*_act_ and less so by *M*_tot_ and *σ* (Fig. 5A). Increasing *k*_act_, *N*_nw,cell_, and *k*_act_*×A*_act_ by a factor two or *k_A_*, *k_B_* by 1.5-fold reduced the activation loss by 6.4 ± 0.9 mV, 6.3 ± 1.3 mV, 6.4 ± 0.9 mV, and 3.9 ± 1.6 mV, respectively, while increasing *M*_tot_ or *σ* by a factor of two led to an increase of activation loss by 1.0 ± 1.1 mV and 0.4 ± 0.7 mV, respectively. *k_A_*, *k_B_*, and *N*_nw,cell_ showed similar effects on activation and ohmic resistance losses, but changes in *k*_act_*×A*_act_, *M*_tot_, and *σ* had opposite impacts, with an increase by a factor two of *k*_act_*×A*_act_, *M*_tot_, and *σ* leading to a change in ohmic resistance losses by 0.5 ± 0.7 mV, 2.4 ± 1.9 mV, and −1.6 ± 1.1 mV, respectively (Fig. 5B). In total, *k_A_*, *k_B_*, *k*_act_*×A*_act_, and *N*_nw,cell_ exhibited substantial impact on net available potential, whereas *M*_tot_ and *σ* showed moderate effects, in part due to the counteracting effect on η_act_ and η_om_ for *M*_tot_ and *σ* (Fig. 5A and B). It should be noted that these results are insensitive to changes in the electron conduction constant (*k_D_*) and electric field associated rate constant (*k_EF_*) (Fig. S14 and S15 at https://doi.org/10.6084/m9.figshare.13536086.v2). In agreement with results reported previously (19), we observed no significant difference between simulations with electric field as the sole driving force and simulations with redox gradient as the driving force. Note that changes in these parameters affect not only the overall energetics for the AOM consortium but also the distribution of cell activity. Changes in *M*_tot_, *σ*, and *k*_act_*×A*_act_ alter the shape of cell activity with distance between syntrophic partners, while *k_A_*, *k_B_*, and *N*_nw,cell_ mostly affect the slope of a linear decrease of activity with distance (Fig. S16 at https://doi.org/10.6084/m9.figshare.13536086.v2).

Because several experimentally poorly characterized model parameters impact the magnitude of activity and spatial patterns of modeled electric losses (Fig. 5, Fig. S11 to S13 at https://doi.org/10.6084/m9.figshare.13536086.v2), our work emphasizes important targets for future study and observation, such as an assessment of the number of pili/wire-like structures recently observed to be involved in extracellular electron transfer (EET) for some archaeal/bacterial syntrophic consortia (18, 22). The accurate quantification of these connections is challenging, as not all such structures are necessarily conductive, and most observations are two-dimensional sections through a three-dimensional matrix of extracellular material. However, the extent to which archaeal and bacterial cells are connected is important, because variations in the extent of conductive connections can substantially alter the metabolic activity pattern by influencing both activation loss, *η*_act_ (Fig. 5A), and ohmic resistance loss, *η*_om_ (Fig. 5B), and, hence, the net available potential, η_net_ (Fig. 5C). Halving *N*_nw,cell_ significantly limited the metabolic activity due to the reduced availability of η_net_ (Fig. 5C), in agreement with Storck et al. (27), who reported that decreasing conductive network density (*N*_nw,cell_) by a factor of 10 led to a 60% decrease of electron transport rate. Doubling *N*_nw,cell_ resulted in a homogenous distribution of metabolic activity, similar to the finding in the study by Storck et al. (27), in which the electron transport rate increased slightly for a 10-fold increase in *N*_nw,cell_, suggesting a plateau was reached. Furthermore, while no data on AOM consortium conductivity, *σ*, have been published yet, such measurements have been made in *Geobacter* biofilms (9, 28–31), *Geobacter* pilin nanofilaments (28, 32), Desulfovibrio desulfuricans nanofilaments (33), methanogenic aggregates from anaerobic wastewater reactor (12), and granules from anaerobic bioreactors (34), among others. The conductivity, *σ*, has a significant impact, with a reduction by a factor of 10 to 10^−3^ S m^−1^ drastically reducing the metabolic activity (Fig. S16C at https://doi.org/10.6084/m9.figshare.13536086.v2). By increasing conductivity to 10^−1^ S m^−1^, metabolic activity reached a homogenous spatial distribution, owing to the increased η_net_ at higher conductivity (Fig. 5C).

### Type and strength of syntrophic coupling between archaea and bacteria.

**FIG 6:**
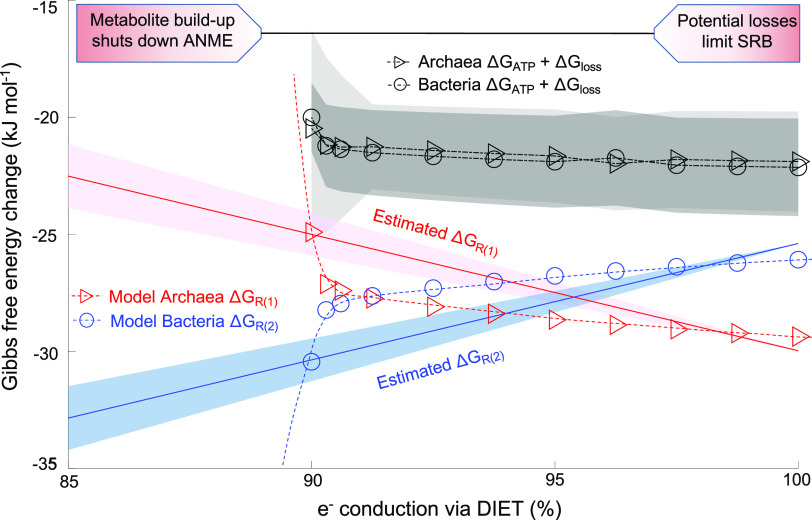
Gibbs free energy change (Δ*G*) against the change of electron conduction via DIET. Circles and triangles represent bacteria and archaea, respectively. Simulations were run at an aggregate radius of 20 μm with baseline parameters. The estimated Δ*G_R_*_(3)_ and Δ*G_R_*_(4)_ were calculated with *CH_4_ =* 4.5 mM, *SO_4_^2−^ =* 28 mM, *HCO_3_*^−^ *=* 2.3 mM, *HS*^−^ *=* 0.1 mM, *MH = M = *5 mM, *pH = *8.2, and *T = *277.15K, with *HCOO*^−^ varying between 0.1 and 100 μM to reflect different intra-aggregate and/or environmental conditions. The light and dark gray-shaded areas represent the resulting 95% confidence intervals for the *Archaea* and *Bacteria*, respectively.

The model was used to assess potential advantages of a mechanism in which electron transport through both DIET and MIET is active. A hybrid DIET-MIET mechanism, as implemented in our baseline simulation, can lead to a higher energy yield than electron transfer by DIET alone, as it allows for more balanced microbial energetics for both syntrophic partners. The conditions for sulfate-reducing bacterial cells were slightly more energetically favorable, with a 92.5% DIET/7.5% MIET hybrid metabolism (Fig. 6 and Fig. S2 at https://doi.org/10.6084/m9.figshare.13536086.v2), with *ΔG_R_*_(4)_ of −26.1 kJ mol^−1^ for 100% DIET versus −27.3 kJ mol^−1^ for a model with mixed DIET and MIET (specific parameters included *CH_4_* = 4.5 mM, *SO_4_^2−^* = 28 mM, *HCO_3_*^−^ = 2.3 mM, HS^−^ = 0.1 mM, *HCOO*^−^ = 1 μM, *MH = M *=* *5 mM, *pH = *8.2, and *T = *277.15K). As a consequence of this difference in reaction energetics, bacterial activity in the 100% DIET simulation decreases more rapidly with separation distance (Fig. S2 at https://doi.org/10.6084/m9.figshare.13536086.v2) than our baseline 92.5/7.5 hybrid model.

Simulations with chemical conditions that vary spatially at rates matching those observed in the ^15^N FISH-nanoSIMS experiments show that at <90% DIET, methane oxidation shut down due to the buildup of the intermediate electron carrier, leading to a net energy gain [*ΔG_R_*_(3)_ − Δ*G*_loss_] less than the minimum requirement for ATP production (Δ*G*_ATP_). At 100% electron conduction by DIET, archaea were generally active and not limited by the accumulation of reaction products, but the bacteria become susceptible to limitation from voltage losses. Consistent with the simplified thermodynamic calculations (Fig. 6), the model simulations showed a narrow window with approximately 90 to 100% DIET that enabled energetically favorable conditions for both bacterial and archaeal cells (Fig. 6). Importantly, a hybrid mechanism can affect the balance of energy gains between the syntrophic partners, which results in improved energetic conditions for the partner most energetically constrained, thereby benefitting both archaea and bacteria (Fig. 6).

### Potential for decoupling of archaeal and bacterial metabolisms.

We considered metabolic decoupling between the ANME and SRB partners, where the bacteria may use electron donors derived from the external environment rather than be provided the syntrophic partner. We explored the impact of an externally sourced electron donor, *DH*, on bacterial metabolism by loosening the coupling between archaeal and bacterial metabolism (see Appendix A2 in the supplemental material at https://doi.org/10.6084/m9.figshare.13536086.v2). Such decoupling has been observed in thermophilic AOM consortia, where it has been shown that the ANME-1 sulfate-reducing bacterial partner HotSeep-1 can utilize H_2_ and grow independently of ANME (18). As MIET using H_2_ is not thought to be an important form of syntrophic electron transfer (18), detectible hydrogenases are lacking in ANME (35) and SRB (36) genomes recovered from cold seeps, and experimental data demonstrated that excess hydrogen addition does not inhibit AOM activity in sediment incubations and enrichment cultures (37–39); for convenience, we continue to consider formate a soluble electron donor. Formate concentrations in marine sediments range from below the detection limit (0.37 μM) to 10.38 μM in Baltic Sea sediments (40), 2 to 18 μM in northern Gulf of Mexico sediments (41), up to 59.5 μM in Hydrate Ridge sediments (42), 12.1 μM in Aarhus Bay sediments (43), and 36 to 158 μmol/kg in fluid from the Lost City hydrothermal field (44). Thus, simulations were carried out for 1 to 100 μM formate in the environment. Increasing formate from 1 μM to 15 μM led to a significant increase of bacterial activity at the aggregate surface while showing nearly no impact on archaeal cells (Fig. S8A at https://doi.org/10.6084/m9.figshare.13536086.v2). At a lower HCOO^−^ concentration (1 μM), bacterial cells exhibited a slight shortage of HCOO^−^ supply away from the archaeal-bacterial interface (Fig. S8B). At high formate concentrations (>15 μM), carrying out archaeal CH_4_ oxidation could become thermodynamically unfavorable due to the accumulation of HCOO^−^ (not shown). Noticeably, the Gibbs free energy change for sulfate reduction [Δ*G_R_*_(4)_] significantly decreased from ∼-27.5 kJ mol^−1^ to −30.05 kJ mol^−1^ when changing formate from 1 μM to 15 μM (Fig. S8C), leading to a significant increase of bacterial thermodynamic constraint *F_T_* from 0.35 to 0.7 at the aggregate surface, while no significant changes were observed for archaea (Fig. S8D). Notably, the increased formate from 1 μM to 15 μM did not significantly impact the total flux of HCOO^−^, although an increase of HCOO^−^ concentration within consortium was observed (Fig. S8B).

### Conclusions.

We report on the metabolic activity distribution of individual cells in a large AOM consortium using FISH-nanoSIMS. A decline in cell activity with the increasing distance from the archaeal-bacterial interface was observed in a section through the center of the aggregate, cut approximately normal to the ANME-SRB interface. These results provide the first quantitative assessment of the growth penalty that exists over large separation distances between these syntrophic partners, an effect that is not apparent in small or well-mixed aggregates (17, 19). A reactive transport model accounting for thermodynamic limitations on cell metabolism, as well as activation and ohmic resistance losses in the exchange of electrons between syntrophic microorganisms, successfully reproduced these novel observations. Direct interspecies electron transfer makes the observed spatially distributed cell activity possible, where at larger distances ohmic losses are predominantly responsible for constraining the interspecies syntrophic partner distance within <30 μm. The process-based model also revealed possible advantages of a hybrid DIET-MIET mechanism, allowing for balanced microbial energetics for both syntrophic partners but opening up the potential for decoupling of the sulfate-reducing bacterial partner from the methanotrophic archaea by utilizing electron donors from the environment. While this points to the possible benefit of versatile and adaptable use of diverse electron donors and modulating association strengths, the nature of such small redox-active molecules acting as electron shuttles remains unknown. Future work will help us answer these mechanistic questions by a careful comparison of ANME and SRB genomic potential and expression with their cellular activity patterns.

## MATERIALS AND METHODS

### Experimental data. (i) Sample collection.

Methane seep sediments covered with white bacterial mats were collected from Jaco Scar, off Costa Rica, at 1,811-m water depth (lat 9.1163, long −84.8372). Samples were collected by push core (PC6) during dive number AD4912 on 27 May 2017 by DSV *Alvin*, launched from R/V *Atlantis* on research cruise AT37-13. The sediment core was processed shipboard into 3-cm-depth horizons that were placed in separate Whirl-Pak bags and stored under anoxic conditions in a large sealed Mylar bag flushed with Ar. These sediments were stored at 4°C until they were returned to the laboratory, where sediments were mixed with N_2_-sparged, 0.2-μm-filtered seawater collected above the sampling site and incubated in anoxic 1-liter Pyrex bottles with a secured butyl rubber stopper supplied with a 100% methane headspace (30 lb/in^2^).

### (ii) Stable isotope probing, incubation, and sampling.

Stable isotope incubation experiments were conducted using slurried sediment from PC6, corresponding to the 3- to 6-cm-depth horizon. Sediment was mixed 1:3 with N_2_-sparged, 0.2-μm-filtered seawater from above the sampling site (28 mM sulfate) and amended with 1 mM NH_4_Cl with 99% ^15^N abundance (Cambridge Isotope Laboratories, Inc.) and incubated at 4°C. Headspace composition was 100% methane at 30 lb/in^2^. After 7 days, subsamples were collected for analysis by first shaking the incubation bottle to resuspend the sediment slurry and then collecting an aliquot using an N_2_-flushed needle and syringe. A volume of 1 ml of sediment was chemically fixed by mixing with 1 ml of 4% paraformaldehyde in 3× PBS and incubated for 1 h at room temperature. Sediments containing AOM aggregates were washed three times with 3× PBS and finally resuspended in 50:50 PBS-ethanol (EtOH) and stored at −20°C.

### (iii) Resin imbedding and FISH staining.

Fifty microliters of fixed sediment slurry in 50:50 PBS-EtOH was mixed with 750 μl PBS in a 2-ml microcentrifuge tube and sonicated on ice with a microtip sonication probe (Branson), 3× for 10 s at setting 3 (8 W). Aggregates were separated from sediment particles by density gradient centrifugation by underlaying the sonicated liquid with 1 ml of Percoll and spinning at maximum speed for 30 min in a tabletop microcentrifuge at 4°C. The top aqueous layer containing concentrated aggregates was removed and pelleted by spinning at 10,000 × *g* at room temperature for 1 min. The pellet was gently removed and immobilized in molten 3% noble agar in PBS. Once solidified, agar was trimmed to a small cube around the pellet and imbedded in glycol methacrylate (Technovit 8100) resin by following the manufacturer’s protocol. Semithin section (1 to 2 μm thick) were cut using a microtome and deposited on water droplets on polylysine-coated slides with Teflon-lined wells (Tekdon, Inc.). FISH hybridization on thin sections was conducted as described previously (17). ANME-2b-specific probe ANME-2b-729 with a dual 3′/5′ Cy3 label (45) and a universal bacterial probe EUB338mix (EUB338, -II, and -III) labeled with fluorescein isothiocyanate (FITC) were used at 35% formamide concentration (supplied by Integrated DNA Technologies). Sections were counterstained with 4′,6-diamidino-2-phenylindole (DAPI) (5 μg/ml) in CitiFluor mounting medium and fluorescently imaged with a fluorescence microscope (Elyra 7; Zeiss) at ×100 magnification (Plan-APOCHROMAT 100× objective).

### (iv) NanoSIMS.

Sections were rinsed with deionized water to remove DAPI and mounting medium, and then glass slides were scored with a diamond scribe, broken, and filed to fit into the nanoSIMS sample holder. Sections and slide fragments were sputter coated with 40 nm of gold (Cressington). Areas containing aggregates of interest were presputtered using a primary cesium ion beam at 90 pA (D1 = 1) until ^14^N^12^C^−^ ion counts stabilized (∼5 min). NanoSIMS images were acquired in 10-μm by 10-μm rasters with 128 by 128 pixels with 0.3 pA (D1 = 3, ES = 3) Cs^+^ ion beam with a 12-ms/pixel dwell time. Between 20 and 30 10-μm by 10-μm acquisitions were tiled across the aggregate with approximately 2-μm overlap, and the data were manually stitched together postanalysis to create final data products. In addition to the new FISH-nanoSIMS data generated for this study, we also incorporated select nanoSIMS data as a point of comparison from published studies with similar experimental designs (17, 46). Regions of interest (ROIs) consisting of individual archaeal and bacterial cells within a consortium were identified and segmented (outlined) by hand using the nanoSIMS ^14^N^12^C^−^ ion images. Archaeal or bacterial identities for each cell were assigned based on comparison of the nanoSIMS image to the corresponding FISH image. Distances between cells were calculated based on the centroid of each segmented cell in MATLAB.

### (v) Cell-specific activity calculation.

Growth rates were calculated from nanoSIMS data by (47)

(1) $$\mu =\frac{-\text{ln}\left(1-\frac{F_{\text{final}}-F_{\text{nat}}}{F_{\text{label}}-F_{\text{nat}}}\right)}{T_{\text{incub}}}$$

where *μ* is the growth rate (encompassing both cell maintenance and generation of new cells), *T*_incub_ is the length of the incubation (7 days), *F*_label_ is the labeling strength of the nitrogen source provided, N15H4+N14H4++N15H4+, *F*_final_ is the nanoSIMS measurement, and *F*_nat_ = 0.0036 is the natural ^15^N abundance. The cell-specific metabolic rates (in mol CH_4_ cell^−1^ day^−1^) were calculated as

(2) $$R_{\text{obs}}=\text{μ}\cdot \rho \cdot B_{\text{cell}}/Y_{\text{CH4}}$$

where *ρ* is the g cell dry weight per m^3^, *B*_cell_ is the cell density in m^3^ per cell, and *Y*_CH4_ is the growth yield in g cell dry weight per mol CH_4_ oxidized. See Table 1 for values and sources.

**TABLE 1:** Summary of parameters used in model implementation

Category and symbol^*a*^	Value	Unit	Description^*b*^	Baseline value	Reference and/or note
Kinetics and thermodynamics					
*k_A_*	10^−13^–10^−17^	m^3^ cell^−1^ day^−1^	Archaeal rate constants	4 × 10^−16^	Estimated from 19
*k_B_*	10^−13^–10^−17^	m^3^ cell^−1^ day^−1^	Bacterial rate constants	4 × 10^−16^	
*K_m_^CH4^*	1–20	mM	Half saturation constant for methane	7	65
*K_m_*^$SO_{4}^{\mathrm{2-}}$^	1–10	mM	Half saturation constant for sulfate	5	66
*f_D_*	0–4		Fraction of electron conduction via MIET	0.4	Estimated from Rxn(3) and Rxn(4)
*f_M_*	0–8		Fraction of electron conduction via DIET	7.4	
*χ*	1		No. of ATP molecules synthesized per reaction	1	20
*η*_ATP_	0.013	V	Potential related to the energy required to synthesize ATP	0.013	Calculated using *η*_ATP_ = −Δ*G*_ATP_/nF
*ΔG*_ATP_	–10	kJ mol^−1^	Energy required to synthesize ATP	−10	56, 57
*R*_gas_	8.314	J K^−1 ^mol^−1^	Gas constant	8.314	
*F*	96,485.3	C mol^−1^	Faraday constant	96,485.3	
*T*	277.15	K	Incubation temp	277.15	Measured
*n*	8		No. of electrons transferred per reaction	8	Calculated from Rxn(3) and Rxn(4)
*k^o^_H_*	0.0014	mol kg^−1 ^bar^−1^	Henry's law constant for methane solubility in water at 298.15 K	0.0014	67
*d*(*ln*(*k_H_*))/*d*(1/*T*)	1,600	K	Henry's law temp dependence constant for methane	1600	
*k_H_*(*T*)	0.0021	mol kg^−1 ^bar^−1^	Henry's law constant for methane solubility in water at *T* = 277.15 K	0.0021	Calculated
*ρ_SW_*	1.03 × 10^3^	kg m^−3^	Density of seawater	1.03 × 10^3^	68
Geometry					
*r_A_*	0.4	μm	Radius of archaeal cell	0.4	2, 58, 59
*r_B_*	0.4	μm	Radius of bacterial cell	0.4	
*r*_agg_	5–200	μm	Radius of AOM aggregate	20	This study and 17, 45, 69
*r_env_*	12.5–500	μm	Radius of environment surrounding aggregate	50	Imposed
*N*_ANME_	Varied	cells	No. of archaeal cells	2.68 × 10^6^	Calculated using consortium vol/cell vol
*V*_agg_	Varied	m^3^	Volume of aggregate	3.35 × 10^−14^	Calculated using *V*_agg_ *=* 4/3π *r*_agg_^3^
Cell-specific activity					
*μ*	Varied	Day^−1^	Cell growth rate		Calculated using equation 1
*ρ*	4.8 × 10^5^	g cell dry wt per m^3^	Biomass density of cells	4.8 × 10^5^	17
*B*_cell_	2.68 × 10^−19^	m^3^ per cell	Cell density	2.68 × 10^−19^	Calculated using B_cell_ = 1/cell vol
*Y*_CH4_	0.2–0.72	g cell dry wt per mol CH_4_ oxidized	Growth yield for archaeal cells	0.65	26
*Y*_$SO_{4}^{\mathrm{2-}}$_	0.1–1	g cell dry wt per mol SO_4_^2−^ reduced	Growth yield for bacterial cells	0.55	Imposed
*T*_incub_	7	Days	Length of the incubation	7	Measured
*F*_label_	1		Labeling strength of ^15^N	1	Measured
*F*_nat_	0.0036		Natural abundance of ^15^N	0.0036	47
*F*_final_	varied		Single-cell nanoSIMS measurement		Measured
Electron conduction					
*M*_tot_	0.01–100	mM	Concentration of redox molecules	10	Estimated from 70
*k_D_*	10^−5^–10^5^	m^4^ mol s^−1^	Rate constant of electron transport on conductive pili or matrix	10^5^	Estimated from *D_M_ = k_D_M*_tot_ *δ*
*k_EF_*	10^−9^–10^5^	m^4^ mol s^−1^	Electric field rate constant	10^−5^	Estimated
*k*_act_	2.5 × 10^−10^–10^−7^	m s^−1^	Activation loss rate constant	2 × 10^−9^	Estimated
*k*_nw_	1,017–1,020	mol^−1^	Constant associated with conductive network	1.2 × 10^19^	Estimated
*δ*	0.7	nm	Redox molecules spacing width	0.7	71
*σ*	10^−4^–10^−1^	S m^−1^	Conductivity of conductive pili or matrix	10^−2^	9, 12, 28–34
*β*	0.5		Charge transfer coefficient	0.5	27
*N*_nw_	10^5^–10^8^		Total conductive connections in an aggregate	4 × 10^6^	Calculated using *N*_nw,cell_ = *N*_nw_/*N*_ANME_
*N*_nw,cell_	1–1,000		No. of connections per cell	64	Estimated; 27
*d*_nw_	4	nm	Diameter of a single pilus	4	28
*A*_nw_	1.26 × 10^−17^	m^2^	Cross-section area of a single pilus	1.26 × 10^−17^	Calculated using *A_nw_ = π (d_nw_/2)^2^*
*A*_act_	10^−14^–10^−12^	m^2^	Redox active surface area per cell, 10% of the cell surface area	2 × 10^−13^	Calculated; 27

a Aqueous diffusion coefficients: *D*_CO2_ = 1.91 × 10^−9^ m^2^ s^−1^, *D*_CO3_ = 1.19 × 10^−9^ m^2^ s^−1^, *D*_H+_ = 6 × 10^−9^ m^2^ s^−1^, *D*_OH_ = 5.27 × 10^−9^ m^2^ s^−1^, *D*_B(OH)4_ = 9.56 × 10^−10^ m^2^ s^−1^, *D_HCOO-_* = 4.9 × 10^−10^ m^2^ s^−1^, *D_HCOOH_* = 1.516 × 10^−9^ m^2^ s^−1^, *D_HS_* = 1.19 × 10^−9^ m^2^ s^−1^, *D_CH4_* = 9.95 × 10^−9^ m^2^ s^−1^, *D_${\mathrm{SO}}_{4}^{\mathrm{2-}}$_* = 6.37 × 10^−10^ m^2^ s^−1^. Fixed concentration boundary conditions are imposed for all chemical species at the outer domain boundary except for MH, for which no flux condition is imposed at the aggregate surface. Boundary conditions are set to 0.1 mM HS^−^, 2.3 mM HCO_3_^−^, pH 8.2, 28 mM *SO*_4_^2−^, 4.5 mM CH_4_, 10 μM HCOO^−^.

b Henry's law constant for methane solubility in water, *k_H_*(*T*), is determined to be 0.0021(mol kg^−1 ^bar^−1^) using *k_H_*(*T*) = *k*°*_H_* exp(*d*(ln(*k_H_*))/*d*(1/*T*) ((1/*T*) − 1/(298.15 K))), where *k*°*_H_* is Henry's law constant for solubility in water at 298.15 K (mol kg^−1 ^bar^−1^) and *d*(ln(*k_H_*))/*d*(1/*T*) is the temperature dependence constant (*K*) (67). The concentration of CH_4_ in incubation medium then can be derived using *[CH_4_] = p_CH4_k_H_(T)ρ_SW_*, where *p_CH4_* is the CH_4_ pressure (bar) and *ρ_SW_* is the density of incubation medium.

### Modeling approach.

Electron transfer between archaea and bacteria was implemented as a mixed DIET-MIET mechanism where electrons from the oxidation of methane are captured by either redox-active molecules (*M* in oxidized form and *MH* in reduced form) that conductively connects archaeal and bacterial partners or by intermediate form (*DH*), which can exchange between the syntrophic partners by diffusion. This highly simplified description minimizes model complexity, reflecting the limited knowledge on the kinetics of the processes part of EET, and is captured by reactions 1 and 2 [Rxn(1) and Rxn(2), respectively]

Rxn(1) $$CH_{4}+H_{2}O+f_{M}M+f_{D}D\to f_{D}DH+f_{M}MH+H^{+}+{HCO}_{\text{3}}^{-}$$

Rxn(2) $$SO_{4}^{2-}{+H}^{\text{+}}+f_{D}DH+f_{M}MH\to f_{M}M+f_{D}D+{HS}^{-}+f_{D}D+H_{2}O$$

where *f_M_* and *f_D_* represent the fraction of electron transfer via MIET and DIET, respectively, and Rxn(1) and Rxn(2) are the (unbalanced) overall metabolic reactions of archaea and bacteria.

We chose formate (48) as a representative intermediate between ANME and SRB to establish the stoichiometry and thermodynamics, but we recognize evidence that suggests otherwise and note that other small molecules could also be considered the putative intermediates for AOM (19, 20, 37, 48–54). This choice affects the energetics and reaction stoichiometries, but, due to similarities arising from diffusion limitations (19, 20, 55), comparable results are obtained in the context of this study.

For a case where formate is identified as the dissolved electron donor, DH, the reactions [Rxn(3) and Rxn(4), respectively] become

Rxn(3) $$CH_{4}+\left(f_{D}-1\right)HCO_{3}^{-}+f_{M}M\to f_{D}HCOO^{-}+f_{M}MH+H^{+}+\left(f_{D}-3\right)H_{2}O$$

Rxn(4) $$SO_{4}^{2-}+H^{+}+f_{D}HCOO^{-}+f_{M}MH\to f_{M}M+HS^{-}+f_{D}HCO_{3}^{-}+\left(4-f_{D}\right)H_{2}O$$

where *f_M_* ∈ [0,8] and *f_D_* = (8-*f_M_*)/2 ∈ [0,4], with *f_M_* = 8 and *f_D_* = 0 in the absence of MIET.

### (i) Rate expression.

Cellular metabolic rate and response can be captured by (56, 57)

(3) $$R^{X}=F_{k}^{X}F_{T}^{X}$$

where $F_{k}^{X}$ represents the reaction kinetics of reaction *X* and is the product of a cell-specific rate constant, *k*, the cell density, *B*_cell_, and the dependence on substrate availability (19):

(4) $$F_{k}^{R1}=k_{A}B_{A}\frac{{CH}_{4}}{K_{m}^{{CH}_{4}}+{CH}_{4}}M$$

(5) $$F_{k}^{R2}=k_{B}B_{B}\frac{SO_{4}^{2-}}{K_{m}^{SO_{4}^{2-}}+SO_{4}^{2-}}MH$$

The thermodynamic factor $\left(0\leq F_{T}^{X}\leq 1\right)$ reflects that there must be sufficient free energy available from the reactions to fuel ATP synthesis and cell maintenance and is given by (56, 57)

(6) $$F_{T}^{X}=\text{max }\left(0,\;1\;-\text{ exp }\left(-\mathrm{nF}\frac{\eta_{\text{net}}^{X}-\eta_{\text{ATP}}\;}{\chi R_{\text{gas}}T}\right)\right)$$

where *n* is the number of electrons per reaction, *χ*, the number of ATP synthesized per reaction, is set to 1 (20), *R*_gas_ is the universal gas constant (8.314 J K^−1 ^mol^−1^), and *T* is temperature (277.15 K). *η*_ATP_ represents the potential related to the energy required to synthesize ATP by *η*_ATP_ = −Δ*G*_ATP_/*nF*, where *F* is the Faraday constant and Δ*G*_ATP_ = −10 kJ mol^−1^ (56, 57). The net available potential is given by

(7) $$\eta_{\text{net}}^{\text{X}}=\eta_{\text{rxn},X}-\eta_{\text{act}}-\eta_{\text{om}}$$

where *η*_rxn,_*_X_* is calculated from the Gibbs free energy, Δ*G_X_*, of reaction for archaea [*X = R*(1)] and bacteria [*X = R*(2)], and *η*_act_ and *η*_om_ are the voltage losses associated with activation and ohmic resistance, respectively. Here, we expand our earlier work (19) by taking into account ohmic resistance and activation loss that ultimately limit metabolic activity away from the archaeal-bacterial interface.

Activation loss describes the energetic loss occurring during the electron transfer between cell and conductive pili/matrix. The voltage drops associated with the electron conduction between *M* and *MH* can be described by the Butler-Volmer equation assuming a one-step, single-electron transfer process (27). The activation loss, *η*_act_, is related to the current density:

(8) $$\frac{I}{N_{\mathrm{nw}}}\;=\;FA_{\mathrm{act}}k_{\mathrm{act}}M_{\mathrm{tot}}(exp\left(\frac{\left(1-\beta \right)F}{R_{gas}T}\eta_{\mathrm{act}}\right)-exp\left(\frac{-\beta F}{R_{\mathrm{gas}}T}\eta_{\mathrm{act}}\right))$$

where *I* is the current produced by methane oxidation (*I* = *f_M_R_1_N*_ANME_*F*, where *R_1_* is the methane oxidation rate in fmol cell^−1^ day^−1^, *N*_ANME_ is the number of archaeal cells, and *F* is the Faraday constant), *A*_act_ is the redox active surface area in m^2^ per cell (27), *k*_act_ is the activation loss-associated constant in m s^−1^, *β* is the charge transfer coefficient, and *M*_tot_ is the concentration of electron-carrying molecules (*M*_tot_ = [*M*] + [*MH*]). *N*_nw_ is the total conductive connections within an AOM consortium and can be described as *N*_nw_ *= M*_tot_*V*_agg_*k*_nw_, where *V*_agg_ is the volume of consortium and *k*_nw_ is the constant associated with conductive network. Conductive network density can be described as *N*_nw,cell_ *= N*_nw_*/N*_ANME_.

The ohmic loss results from electronic resistance to the flow of electrons through the conductive pili/matrix. The corresponding voltage drop is proportional to current density and is given by (27)

(9) $$\eta_{\mathrm{om}}=\frac{R_{\mathrm{nw}}I}{N_{\mathrm{nw}}}=\frac{d}{\sigma A_{\mathrm{nw}}}\frac{f_{M}R_{1}N_{\mathrm{ANME}}F}{M_{\mathrm{tot}}V_{\mathrm{agg}}k_{\mathrm{nw}}}$$

Here, *R*_nw_ is electrical resistance (Ω), which can be further described as *d/*(*σA*_nw_), where *σ* is the electrical conductivity of pilus (S m^−1^), *d* is the distance from archaeal-bacterial interface, and *A*_nw_ is the cross-section area of a single pilus.

Several of the above-described parameters are poorly constrained experimentally, including the characteristics and concentration of redox active molecules (*M*_tot_), the conductive network density (*N*_nw,cell_), its conductivity (σ), and the various constants (*k_A_*, *k_B_*, *d*, and *k*_act_). Other physiological parameters, such as *A*_act_, are highly tunable by the cell (27). Thus, it should be noted that the same modeled activity levels and patterns can be achieved for different combinations of these parameters. For instance, decreasing *N*_nw,cell_ 10-fold can be counterbalanced by increasing conductivity and cell redox active factor, *k*_act_×*A*_act_, by a factor of 10, as is evident from the expressions for activation loss (equation 8) and ohmic resistance (equation 9). To deal with these compensating effects, we identified the key combined parameters of the system and varied those in our simulations. The equations listed above are sensitive to changes in the combined independent parameters, the maximum metabolic activities, *k_A_B_A_*, and *k_B_B_B_*, the maximum cell-specific current, *FA*_act_*k*_act_*M*_tot_, the resistance *d/*(*σA*_nw_), the effective concentrations, $\frac{CH_{4}}{K_{m}^{\mathrm{CH}4}}$ and $\frac{SO_{4}^{2-}}{K_{m}^{SO_{4}^{2-}}}$, where *CH_4_* and $SO_{4}^{\mathrm{2-}}$ should be interpreted as the background environmental concentrations, and the activation parameters, $\frac{\beta \eta_{\text{act}}}{R_{\text{gas}}T}$ and *n* $\frac{\eta_{\text{net}}^{\text{X}}-\eta_{\text{ATP}}\;}{\chi R_{\text{gas}}T}$.

### (ii) Implementation.

A spherical AOM aggregate was implemented at the center of a domain that represents the surrounding environment with a radius 2.5× that of the aggregate (*r*_agg_). The spatial distribution of archaea and bacteria in the aggregate (Fig. 1A) was set to reflect the distribution patterns observed in the nanoSIMS analysis (Fig. 1B). A specific cell ratio of 1:1 was set to archaea and bacteria, with the same radii of 0.4 μm for both archaeal and bacterial cells (2, 58, 59). It is acknowledged that different AOM aggregates may have different cell radii and biovolumes (60), which would impact the estimates of cell-specific rates of the model results reported below.

The concentration fields of *CH*_4_, *HCO*_3_^−^, *CO*_2_(aq), *CO*_3_^2−^, *SO*_4_^2−^, *HS*^−^, *H*^+^, *OH*^−^, *HCOO*^−^, *HCOOH*, and *B*(*OH*)_4_^−^ were simulated subject to diffusive transport and reaction, with aqueous diffusion coefficients listed in Table 1. The concentrations at the outer domain boundary were set to fixed concentrations reflecting environmental conditions (Table 1), which were also used as initial conditions. The distribution of *MH* depends on metabolic rate and electron hopping on conductive pili or matrix. This can be expressed as (61, 62)

(10) $$\frac{\partial \mathrm{MH}}{\partial t}=\;\phi f_{M}R^{X}+\nabla \cdot \left(D_{M}\nabla \left[\mathrm{MH}\right]\right)+\nabla \cdot J_{\mathrm{EF}}$$

where *D_M_* = *k_D_M*_tot_ *δ* is an effective diffusion coefficient (61, 62) that depends on the electron conduction constant (*k_D_*), the distance between two redox-active molecules (δ), and the concentration of electron-carrying molecules, and ∇·*J_EF_* reflects the electron transfer rate driven by a local electric field adapted from (61, 62). This flux is given by $J_{\mathrm{EF}}=k_{\mathrm{EF}}\left[M\right]\left[\mathrm{MH}\right]\left(e^{\frac{\mathrm{\beta FE\delta}}{R_{\text{gas}}T}}-e^{-\frac{\left(1-\beta \right)FE\delta }{R_{\text{ga}s}T}}\right)$, where *k_EF_* is the electric field associated rate constant and *E* is the electric field strength (61, 62).

Acid-base reactions govern the speciation of cell surface-associated immobile carboxy (*R-COOH*) and amino groups (*R-NH*_2_). We considered the dissolved inorganic carbon (DIC) and borate system (63) to quantitatively calculate the carbonate system and dynamically simulate acid-base reactions, using the kinetic implementation described previously (63, 64), with a total boron (*T_B_*) concentration of 0.427 mM and total DIC (*T*_DIC_) of 2.36 mM. Archaeal and bacterial cell density and cell size were held constant in all models, with cell numbers varying with AOM consortia radii. The model was implemented in COMSOL Multiphysics 5.4 (COMSOL Inc., Burlington, MA, USA), and simulations were run to steady state.

Baseline simulations presented below use the parameterization shown in Table 1. It was constrained by literature values where available and chosen to yield rates and rate distributions consistent with the observations.

### Statistical analysis.

Data are represented as means ± standard errors. The statistical difference between the observed and simulated cell-specific activity patterns was assessed by one-way analysis of covariance (ANCOVA) of the slopes of the regression of cell-specific activity versus distance from the archaeal-bacterial interface. *P *values of *<*0.05 were considered statistically significant, whereas *P* values of * >*0.05 indicated no statistical significance for the slopes of the regression lines. The statistical analyses were performed using MATLAB 2018 (MathWorks, Natick, MA, USA).
